# Knee strengthening after orthopedics surgery: A comprehensive analysis using key performance indicators to Guide Physical Therapists

**DOI:** 10.12688/f1000research.156922.2

**Published:** 2025-01-10

**Authors:** Florian Forelli, Jean Mazeas, Mathias Nielsen-Le Roux, Ayrton Moiroux Sahraoui, Nicholas Miraglia, Maciej Bialy, Ismail Bouzekraoui Alaoui, Georgios Kakavas, Andreas Bjerregaard, Maurice Douryang

**Affiliations:** 1 SFMK Lab, Pierrefitte sur Seine, France; 2 Orthopaedic Surgery Department, Clinic of Domont, Ramsay Healthcare, @OrthoLab, Domont, France; 3 Orthosport Rehab Center, Domont, France; 4 Miraglia Private Clinic, Trieste, Italy; 5 Functional Diagnostics Laboratory, Sport-Klinika, Scanmed Sport, Zory, Poland; 6 Institute of Physiotherapy and Health Sciences, The Jerzy Kukuczka Academy of Physical Education, Katowice, Poland; 7 Mohammed VI Faculty of Nursing and Allied Health Professions, Mohammed VI University of Sciences and Health, Casablanca, Morocco; 8 Mohammed VI Center for Research and Innovation, Rabat, Morocco; 9 Department of Physical Education and Sport Science, University of Thessaly, @ErgoMechLab, Trikala, Greece; 10 Fysiotek Spine and Sports Lab, Athens, Greece; 11 Rehabilitation Department, Aspetar Orthopaedic and Sports Medicine Hospital, Doha, Qatar; 12 Department of Physiotherapy and Physical Medicine, University of Dschang, Dscheng, Cameroon

**Keywords:** Knee Strengthening, Rehabilitation, Velocity-Based Training, Range of Motion

## Abstract

This article provides a comprehensive analysis of knee strengthening post-surgery, focusing on key performance indicators (KPIs) essential for recovery and performance enhancement. The study delves into the importance of range of motion (ROM), load management through repetitions maximum (RM) and velocity-based training (VBT), speed variations, repetition schemes for hypertrophy and strength, and the assessment of pain, inflammation, and effusion. Emphasis is placed on dynamic alignment, muscle activation, and rate of perceived exertion (RPE) to tailor individualized rehabilitation programs. The integration of these KPIs ensures a balanced approach, enhancing muscle strength and joint integrity while minimizing injury risk. Regular monitoring and adjustments based on these indicators are recommended to optimize outcomes and ensure sustained progress in knee function and overall mobility.

## Introduction

The knee joint is crucial for many movements in daily life and athletic activities, making strength training essential for performance, injury prevention and rehabilitation. This article delves into the scientific foundations of knee strengthening, focusing on key performance indicators (KPIs) such as range of motion (ROM), load (repetitions maximum and velocity-based training), speed, repetitions (hypertrophy and strength), pain assessment, inflammation and effusion evaluations (stroke test), biomechanics of movement (dynamic alignment of the lower limb), muscle activation (patient’s perception), and rate of perceived exertion (RPE).

The number of KPIs required depends on the patient's overall condition and specific rehabilitation needs.

## Range of motion

ROM is a critical factor in knee health, influencing the efficacy of strengthening exercises. ROM refers to the degree of movement a joint can achieve in a certain plane or direction. For the knee, symmetrical to the uninvolved side in full extension (0 degrees) to full flexion (approximately 135 degrees) is considered normal. Achieving and maintaining this range is vital for joint function and overall mobility. Manske et al. highlights that achieving full ROM post-surgery or injury is necessary for optimal recovery and function. ^1^ Exercise targeting the knee should prioritize regaining and maintaining this range, with progressive loading to ensure joint and muscle adaptation without compromising joint integrity. This is especially important kneeling sports activities or praying position where weighted full knee flexion are required. Consistent monitoring and assessment of ROM can help tailor exercise programs to individual needs, ensuring that any limitations are addressed promptly to avoid long-term deficits.

The recovery ROM is influenced by various factors, including the severity of the injury or surgery, the healing process, and the presence of inflammation or pain. Muscle strength, flexibility, and scar tissue formation also play a significant role, as do external interventions such as manual therapy and adherence to rehabilitation programs. Psychological factors, like fear of movement or low motivation, and overall health conditions, such as age or pre-existing illnesses, can further impact progress.

To enhance ROM, dynamic stretching and flexibility exercises should be incorporated into a knee strengthening regimen. Techniques such as proprioceptive neuromuscular facilitation (PNF) stretching can be particularly effective. PNF stretching involves both stretching and contracting the muscle group targeted, which can help improve both flexibility and strength. Additionally, incorporating low-impact activities such as swimming or cycling can help maintain and enhance ROM without placing excessive stress on the knee joint. Assisted manual technique such a mobilization with movement may potential contribute to increased ROM.

## Load: Repetitions maximum and velocity-based training

The load used in knee strengthening exercises is a fundamental aspect of training effectiveness. Strength training is typically prescribed as load relative percentage to an individual’s maximal ability repetitions maximum (1RM) or measured the maximum weight a person can lift for a given number of repetitions. For knee strengthening, 1RM and 10RM are commonly used benchmarks. The 1RM represents the maximum weight an individual can lift once with proper form, primarily used to assess maximal strength and prescribe training loads in strength-focused programs. In contrast, the 10RM measures the maximum weight that can be lifted ten times consecutively, emphasizing muscular endurance and hypertrophy. While 1RM provides precise data for maximal strength, it poses a higher risk of injury, making it less suitable for rehabilitation. The 10RM, with its lower loads, is safer and more appropriate for progressive recovery programs, offering a balance between load and repetition to promote both strength and endurance. The 1RM test, while effective, may pose a risk of injury if not supervised properly. Conversely, 10RM offers a safer alternative, balancing load and repetition to enhance muscle endurance and strength. Using these metrics allows for the precise prescription of exercise intensity, ensuring that the muscles are adequately stimulated for growth and adaptation. However, estimating a daily RM can be challenging, if the 1RM intended for the particular session or over a given period has changed prior to the session due to daily fluctuations.

Velocity-Based Training (VBT) utilizes the speed of movement to regulate training load. When the load increased an equal relationship in reduction of lifting speed occurs. This loss of velocity continues until 1RM is achieved. This method ensures that athletes train at optimal intensities, maximizing power output while minimizing injury risk. VBT also allows for real-time adjustments, making it highly adaptable to the individual’s performance on any given day. Banyard et al. demonstrated that VBT could optimize strength gains by allowing real-time adjustments to training loads based on movement velocity, ensuring that muscles are consistently challenged while reducing the likelihood of overtraining. ^2, 3^ By incorporating both RM and VBT, a comprehensive training program can be developed that maximizes strength gains while maintaining safety ( Table 1).

**Table 1.** Velocities for different %1RM from an individual load-velocity profile of one player, for back squat and deadlift. ^5^

Load (% 1RM)	Back squat	Deadlift
**40**	1.21	1.0
**45**	1.14	0.94
**50**	1.06	0.87
**55**	0.99	0.81
**60**	0.91	0.75
**65**	0.84	0.69
**70**	0.77	0.62
**75**	0.69	0.56
**80**	0.62	0.5
**85**	0.54	0.44
**90**	0.47	0.37
**95**	0.39	0.31
**100**	0.32	0.25

An effective knee strengthening program should include both heavy resistance training (based on percentage of 1RM). However, coaches should be aware of normal daily fluctuations may change with previously established 1RM. Therefore, by using VBT, daily variability of 1RM should be easier to adjust. Also more experienced and stronger athletes knows how to perform a maximum lift, and therefore their 1RM velocities may be lower than non-experienced lifter. ^4^ While heavy resistance training close to 1RM are focuses on maximizing muscle strength and muscle size, VBT is focusing on ensuring that the knee muscles are trained across a spectrum of intensities and speeds either within the same session or over time ( Table 2).

**Table 2.** An example of an individualized mean set velocity table for the free-weight back squat with each mean set velocity corresponding to a prescribed number of repetitions and intensity range. ^6^

Intensity	Repetitions									
	1	2	3	4	5	6	7	8	9	10
**Maximum**	0.26	0.34	0.38	0.41	0.47	0.51	0.54	0.57	0.6	0.63
**Very heavy**	0.29	0.35	0.39	0.42	0.48	0.52	0.55	0.58	0.61	0.64
**Heavy**	0.35	0.42	0.46	0.49	0.54	0.58	0.61	0.64	0.67	0.69
**Moderately heavy**	0.42	0.49	0.53	0.55	0.6	0.64	0.67	0.7	0.72	0.75
**Moderate**	0.5	0.56	0.59	0.62	0.67	0.7	0.73	0.75	0.78	0.8
**Moderately light**	0.57	0.63	0.66	0.68	0.73	0.76	0.79	0.81	0.83	0.86
**Light**	0.64	0.7	0.73	0.75	0.79	0.83	0.85	0.87	0.89	0.91
**Very light**	0.71	0.76	0.8	0.82	0.86	0.89	0.91	0.93	0.95	0.97

## Speed

**Figure 1.**
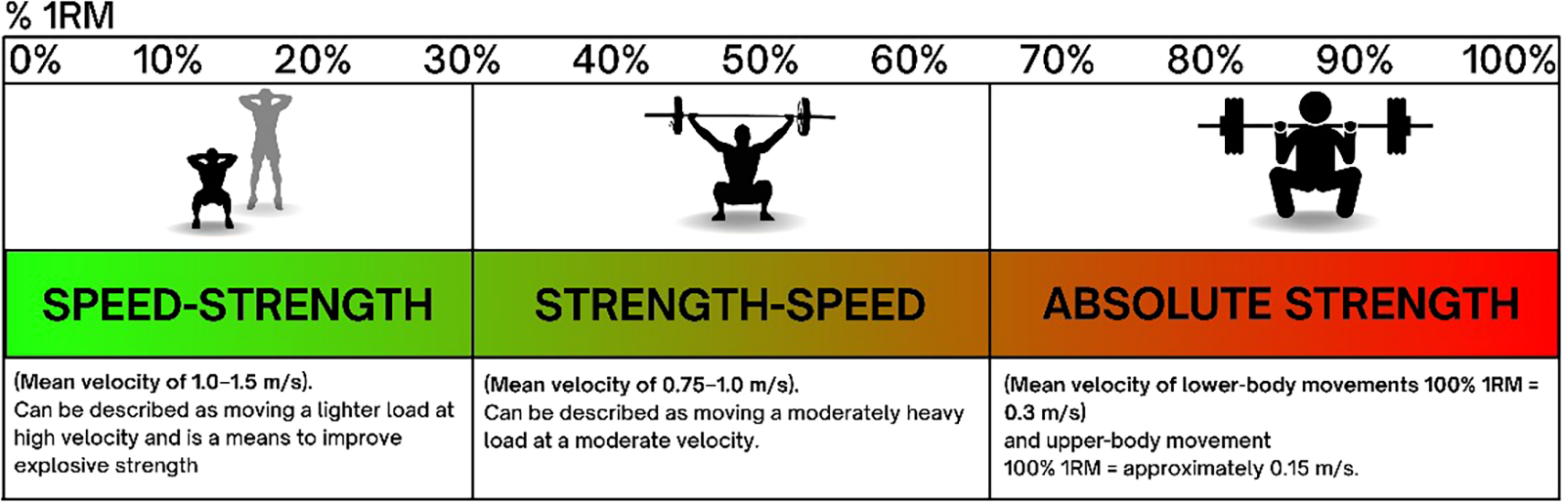
Relationship between %1RM and velocity ranges for different types of muscular strength. ^8^

Speed in knee strengthening exercises refers to the tempo at which movements are performed. Faster speeds can enhance power and neuromuscular coordination, while slower speeds often emphasize muscle hypertrophy and neural drive. Balancing these different tempos can lead to more well-rounded strength development. Behm et al found that varying exercise speed can lead to different adaptations. Fast concentric movements with a high velocity are effective for developing explosive strength 1.3-0.75 m/s, whereas slower eccentric movements 0.75-<0.5 m/s are beneficial for hypertrophy and tendon health. ^7^ Integrating both fast and slow tempos into a training regimen can therefore enhance overall knee function and resilience ( Figure 1).

When designing a knee strengthening program, incorporating speed variations can help supporting the performance goal by appropriate exercise selection towards either more force production or more velocity. From a clinical perspective tracking the same weight being moving with a faster speed over time will also indicate increase performance and equally a decrease in speed can indicate fatigue. Also manipulating load and velocity in plyometric exercises such as jump squats or box jumps can improve explosive power and neuromuscular coordination. Equally, controlled, slow movements like eccentric squats or leg presses will focus more on muscle hypertrophy and tendon health. Implementing VBT training approach ensures comprehensive knee strengthening and functional improvements.

## Repetitions: Hypertrophy and strength

Repetition schemes play a significant role in determining the outcome of a knee strengthening program. Hypertrophy is typically achieved with 6-12 repetitions per set at 60-75% of 1RM, focusing on muscle growth. ^9^ This rep range balances load and volume to promote muscle fiber size increase. On the other hand, strength is developed through lower repetitions (1-6 reps per set at 75-90% of 1RM), geared towards maximizing muscular strength. This approach ensures sufficient load to stimulate neural and muscular adaptations without causing excessive fatigue. Schoenfeld suggests that combining hypertrophy and strength training within a periodized program can lead to optimal muscle growth and strength improvements, offering a balanced approach to knee strengthening. ^10^ Alternating between these repetition schemes can prevent plateaus and maintain continuous progress.

Periodization is a key concept in strength training, involving the systematic planning of training to optimize performance and recovery. By cycling through different phases of hypertrophy and strength training, the knee muscles can be continually challenged and stimulated for growth and adaptation. This approach also helps in preventing overuse injuries and mental burnout, ensuring sustained progress and motivation.

## Pain assessment

Pain assessment is vital in knee strengthening programs, particularly for individuals recovering from injury. Utilizing pain scales help monitor patient discomfort and adjust exercises accordingly. Regular pain assessment can prevent the exacerbation of injuries and guide the progression of exercise intensity. Eitzen et al. emphasize that pain should be monitored to prevent exacerbation of injuries. ^11^ They recommend that patients report pain levels consistently, with modifications to exercises made when pain exceeds a mild threshold. This approach ensures that the training remains within a safe and effective range.

Incorporating pain assessment into a knee strengthening program allows for individualized adjustments based on patient feedback. This can include modifying exercise intensity, volume, or type to ensure that the knee is not being overstressed. Additionally, integrating recovery strategies such as cryotherapy, compression (or compressive cryotherapy in combination), and rest days can help manage pain, reduced medication consumption, and facilitate swelling reduction. ^12^

It is essential to maintain pain levels within a range of 2-3/10 on the pain scale, as this level is optimal for stimulating collagen production. However, if pain exceeds 4/10, the strengthening program must be adapted to prevent overloading the knee and to ensure safe and effective recovery.

## Inflammation and effusion evaluation

Identifying and managing knee effusion may preventing long-term joint damage and ensuring effective rehabilitation. The stroke test is a commonly test used to assess the presence of joint effusion by performing an upward stroke from the medial joint line towards the suprapatellar pouch followed by a downward stroke on the distal thigh from the suprapatellar pouch towards the lateral joint line. If a wave of fluid is observed at the medial knee the test is considered positive. ^13^ The stroke test grades the swelling into five grades from zero where no swelling to 3+ where there are such much fluid that it is not possible to move the effusion out of the medial aspect of the knee. Precautions in exercise progression should be taken when identifying joint effusion to ensure they do not aggravate the knee. ^13^ Regularly conducting the stroke test can help track the knee’s response to training and make necessary adjustments.

Monitoring joint effusion through the stroke test provides valuable information about the knee’s condition and response to exercise for example when returning to running. If effusion is detected, it may indicate an inflammatory response that requires modification of the training program. Rest, anti-inflammatory treatments, and low-impact exercises can be incorporated to allow the knee recover without exacerbating the condition.

## Biomechanics of movement: Dynamic alignment of the lower limb

Proper biomechanical alignment is essential to minimize injury risk and maximize exercise efficiency. Dynamic alignment refers to maintaining proper knee, hip, and ankle positioning during movement. Ensuring correct alignment can prevent common knee injuries and enhance the effectiveness of strengthening exercises. Powers ^8^ discusses the role of dynamic alignment in preventing conditions like patellofemoral pain syndrome. Exercises that enhance lower limb alignment can improve functional outcomes and reduce injury incidence. Emphasizing biomechanical education and motor control or neuromuscular exercises. exercises can significantly improve dynamic alignment during physical activity. Single-leg exercises, such as single-leg squats or lunges, challenge the muscles to stabilize the knee joint and maintain proper alignment in the different planes. Additionally, incorporating balance and vibration training with tools such as balance boards or water bags can enhance proprioception and neuromuscular control.

## Muscle activation: Patient’s perception

Muscle activation refers to the engagement of muscles during exercise. Muscle activation can be directly assessed using electromyography (EMG) or through subjective feedback of the patient. Understanding muscle activation patterns helps in designing exercises that target the intended muscles effectively. Escamilla et al. ^14^ found that exercises like squats and lunges have varying levels of muscle activation in the quadriceps and hamstrings. Incorporating patient feedback on muscle activation can help tailor programs to individual goals. Ensuring optimal muscle activation can enhance the benefits of strengthening exercises by getting quicker results and prevent compensatory movements that may lead to undesired delay in the rehabilitation. An example, if a patient reports low quadriceps activation during squats, adjustments in technique or additional exercises such as leg extensions can be incorporated to enhance activation. This personalized approach ensures that the knee strengthening program is effective and aligned with the patient’s specific goals.

## Rate of Perceived Exertion (RPE)

RPE is a subjective measure of exercise intensity, typically rated on a scale from 1 to 10. It provides insight into how hard an individual feels they are working, allowing for adjustments in training load. Using RPE in conjunction with objective measures such as RM and VBT can create a comprehensive understanding of training intensity. Foster et al. validated the use of RPE in monitoring training intensity. ^15^ They found that RPE correlates well with physiological measures of exercise intensity, making it a useful tool for autoregulating training loads. This allows for a more personalized approach to training, adjusting for daily variations in performance and ensuring consistent progress without overtraining. ^16^ Another way for autoregulating is by using repetition in reserve (RIR). RIR is a measure of how many more repetitions an individual could perform before reaching failure, offering a practical and intuitive way to gauge training intensity and manage fatigue. ^4, 17^

Integrating RPE and RIR into a knee strengthening program helps to balance training load with recovery when VBT is not available. By regularly assessing RPE and RIR, physiotherapists can adjust the intensity and volume of exercises to match the patient’s current condition and prevent overtraining. This dynamic approach ensures that the knee muscles are continuously challenged while allowing for adequate recovery, optimizing long-term progress.

## Conclusion

Strengthening the knee is a multifaceted process that requires attention to various KPIs. When designing individualized knee strengthening programs the focus should be on manipulate on ROM, load, velocity, repetitions, pain assessment, biomechanics, muscle activation, and RPE/RIR. These principles, backed by scientific evidence, ensure that knee strengthening not only enhances performance but also mitigates injury risks as well as long-term joint health.

The integration of clear and well communicated KPIs creates a comprehensive framework for knee strengthening, ensuring that all boxes are checked if any issue to the knee arise during the rehabilitation. Regular assessment and adjustment based on these KPIs allow for personalized and effective training programs that cater to individual needs and goals.

### Ethics and consent

Ethical approval and consent were not required.

## Data Availability

No data are associated with this article.
