# Supplementary material for: Knee strengthening after orthopedics surgery: A comprehensive analysis using key performance indicators to Guide Physical Therapists
Source: F1000Res. 2025 Jan 10;13:1118. Originally published 2024 Oct 3. [Version 2] doi: 10.12688/f1000research.156922.2 (PMC12123294; doi:10.12688/f1000research.156922.2)
Supplement: Peer review history

# Reviewer report: Reviewer response for version 2

**DOI:** 10.5256/f1000research.176200.r385806

**Published:** 2025-06-12

**Authors:** Ali Mohammadi

**Affiliations:** 1 University of Eastern Finland, Kuopio, California, Finland; 2 University of California Davis Health System (Ringgold ID: 70083), Sacramento, California, USA

The manuscript offers an insightful and detailed examination of knee strengthening following orthopedic surgery, utilizing clearly outlined Key Performance Indicators (KPIs). The authors effectively explore critical elements such as range of motion (ROM), load management through Repetitions Maximum (RM) and Velocity-Based Training (VBT), and the importance of exercise tempo variations.

 A noteworthy strength of the article is its clear articulation of methodologies including VBT, pain assessment strategies, the stroke test for evaluating inflammation, and maintaining proper biomechanical alignment. The inclusion of practical recommendations, particularly in the form of specific velocity guidelines corresponding to varying exercise intensities and repetition schemes, significantly boosts the article's practical value for clinicians.

 The manuscript is well-supported by relevant and contemporary literature, enhancing its scientific validity and applicability. The conclusions are logically derived from the presented evidence, convincingly linking proposed rehabilitation techniques to improved clinical outcomes.

 To make the manuscript even more practical for clinical use, the authors might consider:

       1. Briefly address clinicians' potential challenges or limitations when implementing these KPIs across diverse rehabilitation scenarios.

        2. Incorporating additional illustrative clinical examples or brief case studies demonstrating the successful application of the outlined KPIs in real-world patient care.

 In conclusion, this article represents a meaningful addition to the field of rehabilitation, offering clinicians clear, evidence-based guidance that can directly improve patient care and recovery outcomes.

Is the topic of the opinion article discussed accurately in the context of the current literature?

Yes

Are arguments sufficiently supported by evidence from the published literature?

Yes

Are all factual statements correct and adequately supported by citations?

Yes

Are the conclusions drawn balanced and justified on the basis of the presented arguments?

Yes

Reviewer Expertise:

Biomechanics, Knee biomechanics, ACL reconstruction, musculoskeletal modeling, soft tissue mechanics, finite element modeling

I confirm that I have read this submission and believe that I have an appropriate level of expertise to confirm that it is of an acceptable scientific standard, however I have significant reservations, as outlined above.

# Reviewer report: Reviewer response for version 2

**DOI:** 10.5256/f1000research.176200.r382429

**Published:** 2025-05-29

**Authors:** Shaikh Nabi Bukhsh Nazir

**Affiliations:** 1 University of Karachi, Karachi, Sindh, Pakistan

This manuscript presents a commendable effort to develop a guide for physical therapists involved in knee rehabilitation following surgery. The content appears to be well-organized and relevant to clinical practice. I did not find any errors in the manuscript, and the information provided seems accurate and useful for professionals in the field.

Is the topic of the opinion article discussed accurately in the context of the current literature?

Yes

Are arguments sufficiently supported by evidence from the published literature?

Yes

Are all factual statements correct and adequately supported by citations?

Yes

Are the conclusions drawn balanced and justified on the basis of the presented arguments?

Yes

Reviewer Expertise:

Knee osteoarthritis and musculoskeletal disorders

I confirm that I have read this submission and believe that I have an appropriate level of expertise to confirm that it is of an acceptable scientific standard.

# Reviewer report: Reviewer response for version 2

**DOI:** 10.5256/f1000research.176200.r358168

**Published:** 2025-01-21

**Authors:** Ismail Laher

**Affiliations:** 1 University of British Columbia, British Columbia, Canada

No further comments

Is the topic of the opinion article discussed accurately in the context of the current literature?

Partly

Are arguments sufficiently supported by evidence from the published literature?

Partly

Are all factual statements correct and adequately supported by citations?

Partly

Are the conclusions drawn balanced and justified on the basis of the presented arguments?

Partly

Reviewer Expertise:

Pharmacology and physiology

I confirm that I have read this submission and believe that I have an appropriate level of expertise to confirm that it is of an acceptable scientific standard.

# Response

**Published:** 2025-01-21

**Authors:** florian forelli

**Affiliations:** OrthoLab, Clinic of Domont, Domont, France

I wanted to take a moment to sincerely thank you for taking the time to review our manuscript. I truly appreciate the effort and care you put into providing your feedback.

 Your comments and suggestions have been extremely valuable in helping us improve the quality of our work

# Reviewer report: Reviewer response for version 1

**DOI:** 10.5256/f1000research.172304.r343352

**Published:** 2024-12-16

**Authors:** Ismail Laher

**Affiliations:** 1 University of British Columbia, British Columbia, Canada

The authors seem not to have identified a target audience that this review aimed at--and this is a major weakness. The article is fragmented and contains many typographical and grammatical errors.  

 How many KPIs are needed for recovery?

 Are the KPIs the same when one see  or both knees are affected?

 Differentiate between 1RM vs 10RM--and explain the differences?

 Why would it be useful to to know "daily RM"?  

 What are the determinants of recovery of ROM?  

 The article is very cryptic. The section on Pain Management is quite weak and adds little value to the article.

Is the topic of the opinion article discussed accurately in the context of the current literature?

Partly

Are arguments sufficiently supported by evidence from the published literature?

Partly

Are all factual statements correct and adequately supported by citations?

Partly

Are the conclusions drawn balanced and justified on the basis of the presented arguments?

Partly

Reviewer Expertise:

Pharmacology and physiology

I confirm that I have read this submission and believe that I have an appropriate level of expertise to confirm that it is of an acceptable scientific standard, however I have significant reservations, as outlined above.

# Response

**Published:** 2024-12-19

**Authors:** florian forelli

**Affiliations:** OrthoLab, Clinic of Domont, Domont, France

The authors seem not to have identified a target audience that this review aimed at--and this is a major weakness. The article is fragmented and contains many typographical and grammatical errors.  

 We changed the title to target the audience: Knee strengthening after orthopedics surgery: A comprehensive analysis using key performance indicators to Guide Physical Therapists

 How many KPIs are needed for recovery?

**Response: **We added at the end of the introduction "The number of KPIs required depends on the patient's overall condition and specific rehabilitation needs."

 Are the KPIs the same when one see  or both knees are affected? 

**Response: **In orthopedic rehabilitation, it is common for only one knee to be operated on; therefore, our focus is primarily on this aspect. We have clarified this point in the title.

 Differentiate between 1RM vs 10RM--and explain the differences?

**​​​​​​​Response: **We added "The 1RM represents the maximum weight an individual can lift once with proper form, primarily used to assess maximal strength and prescribe training loads in strength-focused programs. In contrast, the 10RM measures the maximum weight that can be lifted ten times consecutively, emphasizing muscular endurance and hypertrophy. While 1RM provides precise data for maximal strength, it poses a higher risk of injury, making it less suitable for rehabilitation. The 10RM, with its lower loads, is safer and more appropriate for progressive recovery programs, offering a balance between load and repetition to promote both strength and endurance."

 Why would it be useful to to know "daily RM"?  

**​​​​​​​Response: **We have already written in the text: "However, estimating a daily RM can be challenging, if the 1RM intended for the particular session or over a given period has changed prior to the session due to daily fluctuations."

 What are the determinants of recovery of ROM?  

**​​​​​​​Response: **We added: The recovery ROM is influenced by various factors, including the severity of the injury or surgery, the healing process, and the presence of inflammation or pain. Muscle strength, flexibility, and scar tissue formation also play a significant role, as do external interventions such as manual therapy and adherence to rehabilitation programs. Psychological factors, like fear of movement or low motivation, and overall health conditions, such as age or pre-existing illnesses, can further impact progress.

 The article is very cryptic. The section on Pain Management is quite weak and adds little value to the article.

**​​​​​​​Response: **We added: It is essential to maintain pain levels within a range of 2-3/10 on the pain scale, as this level is optimal for stimulating collagen production. However, if pain exceeds 4/10, the strengthening program must be adapted to prevent overloading the knee and to ensure safe and effective recovery.
